# Cytotoxic effects of ex vivo*-*expanded natural killer cell-enriched lymphocytes (MYJ1633) against liver cancer

**DOI:** 10.1186/s12885-019-6034-1

**Published:** 2019-08-19

**Authors:** Jung-Won Choi, Eui Soo Lee, Se Young Kim, Su Il Park, Sena Oh, Jung Hwa Kang, Hyun Aae Ryu, Seahyoung Lee

**Affiliations:** 1 Institute for Bio-Medical Convergence, College of Medicine, Catholic Kwandong University, Gangneung-si, Gangwon-do 25601 Republic of Korea; 2 IMMUNISBIO Co., Ltd, International St. Mary’s Hospital, Incheon Metropolitan City, 22711 Republic of Korea

**Keywords:** Natural killer-enriched lymphocytes (NKL), Ex vivo-expansion, Liver cancer, Anti-tumor immunotherapy, MY1633, Cytotoxicity

## Abstract

**Background:**

Adoptive transfer of immune cells such as T cells and natural killer (NK) cells has emerged as a targeted method of controlling the immune system against cancer. Despite their significant therapeutic potential, efficient methods to generate adequate numbers of NK cells are lacking and ex vivo-expansion and activation of NK cells is currently under intensive investigation. The primary purpose of this study was to develop an effective method for expansion and activation of the effector cells with high proportion of NK cells and increasing cytotoxicity against liver cancer in a short time period.

**Methods:**

Expanded NK cell-enriched lymphocytes (NKL) designated as “MYJ1633” were prepared by using autologous human plasma, cytokines (IL-2, IL-12 and IL-18) and agonistic antibodies (CD16, CD56 and NKp46) without an NK cell-sorting step. The characteristics of NKL were compared to those of freshly isolated PBMCs. In addition, the cytotoxic effect of the NKL on liver cancer cell was examined in vitro and in vivo.

**Results:**

The total cell number after ex vivo-expansion increased about 140-fold compared to that of freshly isolated PBMC within 2 weeks. Approximately 78% of the expanded and activated NKL using the house-developed protocol was NK cell and NKT cells even without a NK cell-sorting step. In addition, the expanded and activated NKL demonstrated potent cytotoxicity against liver cancer in vitro and in vivo.

**Conclusion:**

The house-developed method can be a new and effective strategy to prepare clinically applicable NKL for autologous NK cell-based anti-tumor immunotherapy.

**Electronic supplementary material:**

The online version of this article (10.1186/s12885-019-6034-1) contains supplementary material, which is available to authorized users.

## Background

Cancer immunotherapy has been an attractive approach for a long time. There have been many attempts using diverse immunotherapeutic modalities with varying clinical results. Especially, adoptive transfer of immune cells such as T cells and natural killer (NK) cells has emerged as a targeted method of controlling the immune system against cancer [1–3]. Together with CD8^+^ cytotoxic T (Tc) cells, NK cells have received considerable attention because of their roles in in vivo immune surveillance which is to destroy infected or transformed cells [4–6].

NK cells play an important role in the innate immune response. They have highly regulated cytolytic capacity that is achieved by the release of perforins and granzymes as well as expression of Fas ligand and tumor necrosis factor (TNF)-related apoptosis-inducing ligand (TRAIL) [7, 8]. Furthermore, they are involved in T-cell recognition of infected cells and interact with macrophages, granulocytes and dendritic cells through secreted cytokines (interferon (IFN)-γ, interleukin (IL)-13, TNF-α and RANTES), chemokines (C-C motif ligand (CCL)-3, − 4 and − 5) and growth factor (granulocyte–macrophage colony-stimulating factor (GM-CSF)) [7, 9]. The biological activity of NK cell is regulated through fine-tuning of activating and inhibitory receptors. Those receptors maintain cytotoxic capacity against tumor cells without harming healthy cells. Many studies have demonstrated the safety and clinical anti-cancer effects of adoptive transfer of NK cells, highlighting the potential of NK cells as an effective cancer immunotherapy [2, 10].

Nevertheless, a major roadblock to the development of NK cell-based therapies is the lack of efficient methods to generate sufficient number of NK cells for clinical application and efficacy. Since ex vivo-expansion and activation of NK cells is proven to be very challenging, development of an optimized method of preparing NK cells for clinical application is vital to the realization of NK cell-based immunotherapies.

To date, NK cells have been expanded from multiple sources, including peripheral blood mononuclear cells (PBMC) [6, 11–16], umbilical cord blood [2, 17, 18] and embryonic stem cells [19]. Various stimuli such as cytokines [16, 20], monoclonal antibodies [13, 14, 21], and allogenic feeder cells [2, 6, 11, 15, 17] also have been used. Additionally, most of the previous NK cell ex vivo-expansion protocol involved culturing the cells following a process of NK-cell sorting through positive and/or negative selection [6, 11, 15–17]. One of the main purposes of NK-cell sorting is to minimize the risk of immune-related adverse complications such as graft-versus-host disease (GVHD), especially in case of allogeneic applications [22]. This is because non-NK immune cells, such as alloreactive T cells, can cause GVHD following allogeneic adoptive cell therapy [23]. Therefore, NK-cell sorting may not be necessary if the intended use of ex vivo expanded NK cells is autologous adoptive cell therapy. Furthermore, skipping the cell sorting step could simplify the NK cell preparation protocol and reduce the production costs of autologous NK cell-based product if such product should be developed [24].

In the present study, as an effort to develop an effective NK cell-based therapeutics for autologous adoptive cell therapy, we tested a relatively simple but effective method for the expansion and activation of NK cell enriched effector cells and examined the cytotoxic capacity of the prepared cells against liver cancer cells.

## Methods

### Human subjects

Human PBMCs and plasma used for the experiment were obtained from the human blood samples of healthy donors recruited at IMMUNISBIO Co., Ltd. All donors provided an informed consent to participate. The study protocol was approved by the Institutional Review Board, Korea National Institute for Bioethics Policy (P01–201706–31-003).​.

### Isolation of PBMC and plasma

Human blood (30 to 60 mL) was collected using vacuum-driven BD Vacutainer Blood Collection Tubes containing heparin (cat no. 367874; BD, Franklin Lakes, NJ, USA). Autologous human PBMCs and plasma were collected from the buffy coats and the upper aqueous phase of blood samples, respectively, by density gravity centrifugation using Ficoll-Paque (cat. no. 17–1440-03, GE Healthcare, Piscataway, NJ, USA). The lymphocyte-containing PBMCs were washed with phosphate buffered saline (cat. no. LB-004-01; WELGENE, Gyeongsan, Korea) and counted using a hemocytometer. The obtained plasma was inactivated for 30 min at 56 °C for future use (culture and expansion of NKL).

### Composition of NKL culture media

The KBM502 (cat no. 16025020; KOHJIN Bio, Sakado city, Saitama, Japan)-based culture medium used to expand and activate the isolated PBMC was supplemented with the following ingredients; 0.5% or 10% autologous human plasma depending on the step, each 2.5 μg of 3 different agonistic antibodies (anti-human CD56 (cat. no. 555513 and clone B159 (RUO); BD Biosciences, San Jose, CA, USA), anti-human CD16 (cat. no. 555403 and clone 3G8 (RUO); BD Biosciences) and anti-human CD355 (cat. no. MAB1850–500 and clone #195314; R&D SYSTEMS, Minneapolis, MN, USA)), and 3 different cytokines (200 ng/mL of IL-2 (cat. no. 653601261; Norvatis, Whippany, USA), 10 ng/mL of IL-12 (cat. no. 200–12; PEPROTECH, Rocky Hill, NJ, USA) and 100 ng/mL of IL-18 (cat. no. B003–2; R&D SYSTEMS)). All the cytokines were from PEPROTECH (Rocky Hill, NJ, USA).

### Expansion and activation of NKL

**Fig. 1:**
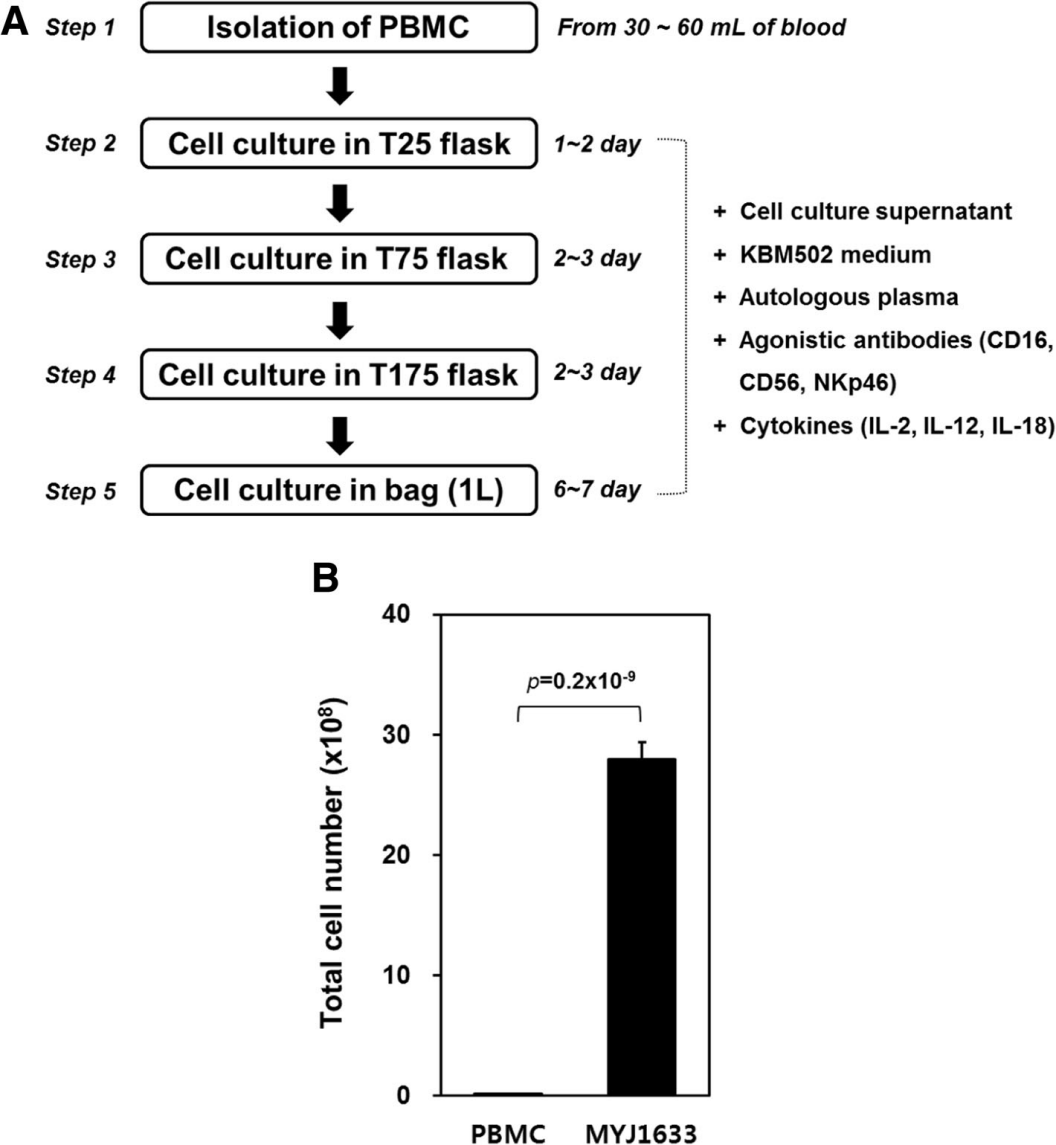
Ex vivo-expansion of MYJ1633. **a** Experimental scheme for ex vivo-expansion of MYJ1633. More details about culture methods of MYJ1633 were described in the Methods section. **b** Comparison of total cell number between PBMC and MYJ1633 from 10 individuals. Significant differences between PBMC and MYJ1633 were determined by Student’s t test. The data represented as mean ± standard error of measurement (SEM)

The isolated PBMCs from the first step were cultured in T25 flask (cat no. 70025; SPL Life Science, Pocheon, Korea) with 10 ml of NKL culture medium containing 10% autologous human plasma for a day or two (second step). The cells and supernatants in T25 flask were then transferred to T75 flask (cat no. 70075; SPL Life Science) and new NKL culture medium containing 10% autologous human plasma was added to the flask to make a final volume of 30 ml (third step). After two or 3 days, the cells and supernatants in T75 flask were transferred to T175 flask (cat no. 71175; SPL Life Science) and 40 ml of new NKL culture medium containing 10% autologous human plasma was added to the flask to make a final volume of 70 ml (fourth step). After two or 3 days, the cells and supernatants in T175 flask were injected into gas-permeable culture bag (cat no. 1602502B; KOHJIN Bio) and 1000 mL of new NKL culture medium containing 0.5% autologous human plasma was added. The cells were cultivated for six or 7 days (fifth step; Fig. 1a). The final product was named after the project (MYJ1633) and the name “MYJ1633” was used to refer the ex-vivo expanded NKL prepared by the house-developed protocol for the rest of the study.

### Flow cytometry

To estimate the cellular composition of the MYJ1633, the cells were incubated with a premixed antibody cocktails in flow cytometry staining buffer containing 1% fetal bovine serum (FBS; Gibco by Life Technologies, Grand island, NY, USA) and 0.09% sodium azide (Sigma-Aldrich, St. Louis, MO, USA). To determine the ratio of NK cells to T cells, 0.25 μg of anti-human CD56 PE-Cyanine7 antibodies (cat. no. 25–0567-42 and clone CMSSB; eBioscience, San Diego, CA, USA), 0.03 μg of anti-human CD16 PE-Cyanine7 antibodies (cat. no. 25–0168-42 and clone CB16; eBioscience) and 0.125 μg of anti-human CD3 APC antibodies (cat. no. 17–0036-42 and clone SK7; eBioscience) were used for the antibody cocktail. For determining the ratio of helper T cells (Th) to cytotoxic T cells (Tc), 0.25 μg of anti-human CD4 FITC antibodies (cat. no. 11–0047-42 and clone SK3 (SK-3); eBioscience) and 0.125 μg of anti-human CD8- PerCP-eFluor®710 antibodies (cat. no. 46–0087-42 and clone: SK1; eBioscience) were used. For detecting different types of receptors expressed, 0.25 μg of anti-human CD56 FITC antibodies (cat. no. 11–0566-42 and clone TULY56; eBioscience), 0.03 μg of anti-human CD16 FITC antibodies (cat. no. 11–0168-41 and clone CB16; eBioscience), 0.125 μg of anti-human CD314 (NKG2D) PE antibodies (cat. no. 12–5879-42 and clone 5C6; eBioscience), 0.25 μg of anti-human CD226 APC antibodies (DNAM-1; cat. no. 338312 and clone 11A8; BioLegend, San Diego, CA, USA), 0.125 μg of anti-human CD336 (NKp44) PerCP-eFluor®710 antibodies (cat. no. 46–3369-42 and clone 44.189; eBioscience), 0.125 μg of anti-human CD355 (NKp46) APC antibodies (cat. no. 17–3359-42 and clone 9E2; eBioscience) and 0.125 μg of anti-human CD94 (NKG2A) APC antibodies (cat. no. 17–5094-42 and clone HP-3D9; eBioscience) were used to stain receptors. The cells were incubated with flow cytometry staining buffer containing appropriate combinations of antibodies for 30 min on ice and washed with PBS twice. For each sample, 100,000 events were captured and data were processed using an Acurri C6 plus flow cytometer (BD Biosciences). Compensation of the spillover between different fluorochromes was performed by using Acurri C6 software according to the user manual provided. Since our unpublished data indicated that more than 95% of CD56^+^ cells were also positive for CD16, an antibody cocktail containing both CD16 and CD56 antibodies were used for detecting CD16^+^ and CD56^+^ cells.

### Enzyme-linked immunosorbent assay (ELISA)

Supernatants were collected after 24 h from cytokine-stimulated and expanded MYJ1633. Interferon (IFN)-γ and tumor necrosis factor (TNF)-α cytokine levels in cell culture supernatants were quantitatively measured using Human IFN-γ ELISA Kit (Abcam; Cambridge, United Kingdom) and Human TNF-α ELISA Kit (Abcam), following the manufacturer’ instructions.

### Cell lines

Hep3B (hepatocellular carcinoma, ATCC® HB-8064), HepG2 (hepatoblastoma, ATCC® HB-8065) and SK-Hep1 (hepatocellular adenocarcinoma, ATCC® HTB-52) cells were purchased form the American Type Culture Collection (ATCC, Manassas, VA, USA). Hep3B and SK-Hep1 were cultured in Dulbecco’s Modified Eagle’s Medium (DMEM; Gibco by Life Technologies) supplemented with 4.5 g/L D-glucose, 10% heat-inactivated FBS, 2 mM L-glutamine (Gibco by Life Technologies), 1 mM sodium pyruvate (Gibco by Life Technologies) and 10 mM HEPES (Gibco by Life Technologies). HepG2 was cultured in Minimum Essential Medium (MEM; Gibco by Life Technologies) supplemented with heat-inactivated 10% FBS, 2 mM L-glutamine, 1 mM sodium pyruvate and 10 mM HEPES. The cells were maintained in a humidified atmosphere of 5% CO_2_ and 95% air at 37 °C.

### Cell viability and cytotoxicity assay

Three different lines of liver cancer cells (Hep3B, SK-Hep1 and HepG2) were seeded at a density of 1 × 10^4^ cells/well in a 96-well plate. After 24 h, MYJ1633 were applied to the liver cancer cells with different effector (E, MYJ1633) to target (T, cancer cells) ratios (2.5:1, 5:1, 10:1 and 20:1). After 24 h of culture, the supernatants were collected, centrifuged to remove unattached MYJ1633 and debris, and were objected to LDH (lactate dehydrogenase) assay [25] to determine the cytotoxicity of MYJ1633 against cancer cells using Cytotoxicity Detection Kit (Takara, Nojihigashi, Kusatsu, Shiga, Japan). The viability of remained (attached) cancer cells after the co-culture with MYJ1633 was measured using Ez-Cytox (DOGEN, Seoul, Korea) [26].

### Xenograft model using SK-Hep1 cell

All experimental procedures for animal studies were approved by the Committee for the Care and Use of Laboratory Animals of Catholic Kwandong University College of Medicine and were performed in accordance with the Committee’s Guidelines and Regulations for Animal Care (CKU 01–2017-008). Male athymic nude mice (Koatech, Pyeoungtaek, Korea) were used at 5 weeks of age. The animals were housed in microisolator cages. To evaluate anti-tumor efficacy of MYJ1633, the animals were subcutaneously bolus injected with SK-Hep1 (3 × 10^6^ cells/animal) in the right flank. After the group assignment (day 0), the animals for the MYJ1633 treated groups were received MYJ1633 (3 × 10^6^ or 6 × 10^5^ cells/200 μl of PBS) via tail vein using a disposable syringe (1 ml, 29G, BD bioscience) at a rate of 2 ml/ml under physical restraint with great care [27]. A mixture of Zoletile (tiletamine/zolazepam, 40 mg/kg) and xylazine (5 mg/kg) was used to anesthetize the animals during handling. MYJ1633 injection was conducted every 7 days from day 1 for 3 times. The volume of tumor was determined by the following formula; width x width x length / 2. The volume of tumor was measured every 3 days on average. At the end of the in vivo study (day 24), and tumors were excised for measurement [28]. Finally, the animals were euthanized via additional i.p injection of Zoletile (30 mg/kg) and xylazine (10 mg/kg).

### Chemical analysis of blood

At the end of the in vivo study, approximately 500 μl of blood was collected from retroorbital venous plexus using capillary tube. The samples were centrifuged at 3000 xg for 15 min to isolate blood serum, and the serum was used to determine the amount of blood alkaline phosphatase (ALP), glutamate oxalacetate transaminase (GOT), glutamate pyruvate transaminase (GPT) and total bilirubin (TBIL) using an automated clinical chemistry analyzer (Fuji Dri-Chem NX500i, FUJI photo film Co., LTD. Tokyo, Japan).

### Immunohistochemistry

To identify human NK cells in tumor mass, mouse monoclonal CD16 antibodies (Santa Cruz Biotechnology, Dallas, TX, USA) were used. In brief, tumor mass sections (24d) were blocked in 2.5% normal horse serum and incubated overnight with the primary antibodies at 4 °C. FITC-conjugated anti-mouse IgG (Jackson Immuno Research Laboratories, West Grove, PA, USA) was used as secondary antibody. Immunofluorescence was detected by a confocal microscope (LSM710; Carl Zeiss Microscopy GmbH, Jena, Germany).

### Statistical analysis

All quantified data are the averages of at least triplicate samples and the error bars represent the SD of the mean. Statistical significance was determined by Student’s t test, and *p* values < 0.05 were considered significant.

## Results

### Experimental scheme and total cell number of MYJ1633 following ex vivo expansion

To preferentially amplify NK cells in PBMCs, blood-isolated PBMCs were cultured in the presence of agonistic antibodies against activating receptors (CD16 and CD56) and natural cytotoxic receptor (NKp44 and NKp46) of NK cells and selected cytokines (Fig. 1a). After 2 weeks of culture, the total cell number of the expanded NKL using our methods increased approximately 140-fold compared to that of initially isolated PBMCs (2 × 10^7^ vs. 2.8 × 10^9^ cells, Fig. 1b). The ex-vivo expanded NKL was designate as “MYJ1633” after a project developing culture protocol.

### Identifying key cell types of MYJ1633 following ex vivo expansion

**Fig. 2:**
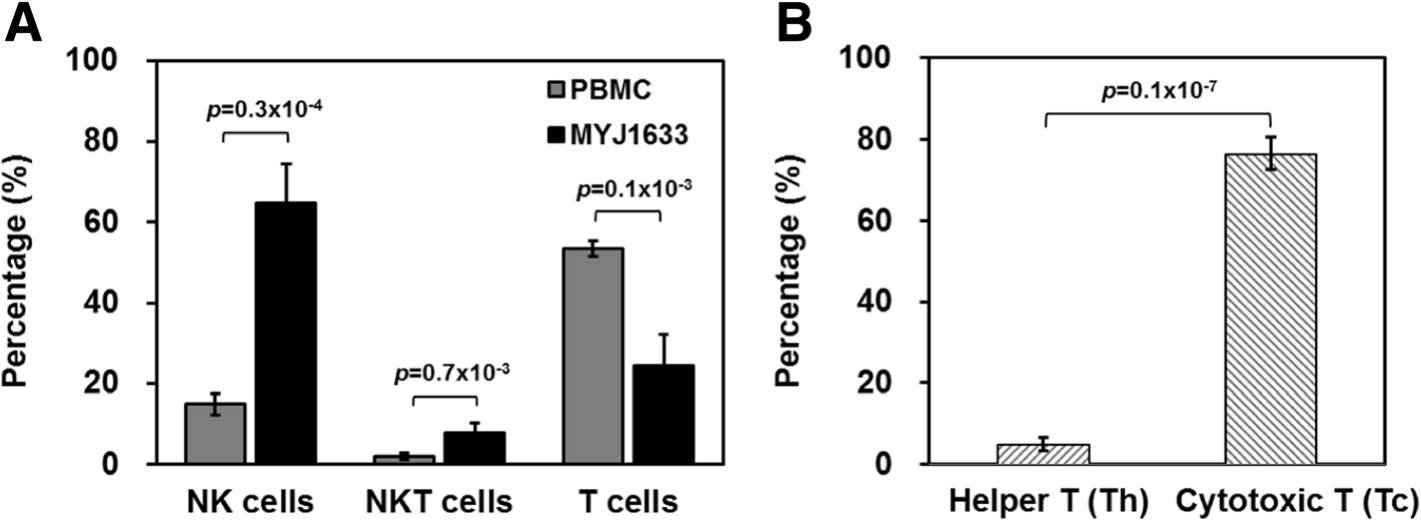
Identification of key immune cell types of MYJ1633 following ex vivo expansion. **a** The distribution of NK cells (CD3^−^CD16^+^CD56^+^), NKT cells (CD3^+^CD16^+^CD56^+^), and T cells (CD3^+^CD16^−^CD56^−^) of freshly isolated PBMCs and MYJ1633 was examined by flow cytometry. **b** Proportion of helper T cells (Th cells; CD4^+^) and cytotoxic T cells (Tc cells; CD8^+^) among CD3^+^ cells of MYJ1633. These data were analyzed from 6 individuals (Additional file 1: Figure S1). Significant differences between groups were determined by Student’s t test. The data represented as mean ± SEM

The proportion of NK cells (CD3^−^/CD16^+^/CD56^+^), natural killer T cells (NKT, CD3^+^/CD16^+^/CD56^+^), and T cells (CD3^+^CD16^−^CD56^−^) in initially isolated PBMCs and MYJ1633 was determined using flow cytometry. In the initially isolated PBMCs, the ratio of CD16^+^/CD56^+^ cells (NK plus NKT cells) to T cells was 0.346, but it increased in MYJ1633 to 3.888 indicating that CD16^+^/CD56^+^ cells were preferentially expanded compared to T cells under the given culture condition. In MYJ1633, the percentage of NK cells (CD3^−^CD16^+^CD56^+^), NKT cells (CD3^+^CD16^+^CD56^+^) and T cells (CD3^+^CD16^−^CD56^−^) were 64.7 ± 9.6%, 7.7 ± 2.5% and 24.4 ± 7.8% of the total cells, respectively (Fig. 2a). Additionally, majority of the T cell population was CD8^+^ cytotoxic T (Tc) cells (76.5 ± 4%) rather than CD4^+^ helper T (Th) cells (4.9 ± 1.7%) in MYJ1633 (Fig. 2b). Analyzed data using flow cytometry in PBMC and MYJ1633 are shown in (Additional file 1: Figure S1).

### Examining receptors of MYJ1633 following ex vivo expansion

**Fig. 3:**
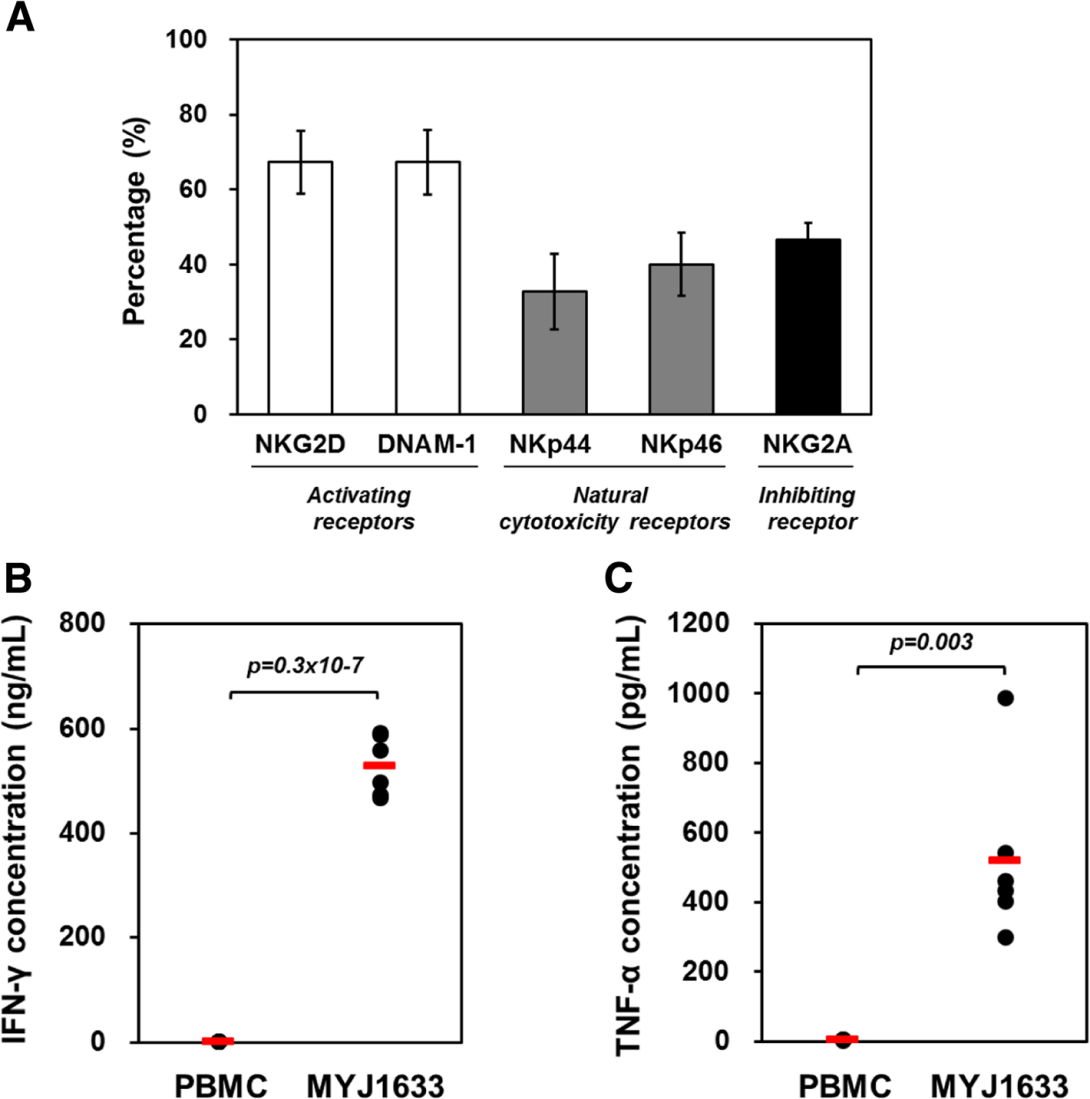
Functional receptor expression and cytokine production of MYJ1633. (**a**) The expression of activating, natural cytotoxicity, and inhibiting receptors on CD16^+^ CD56^+^ MYJ1633 was determined by using flow cytometry. The data was analyzed from 6 individuals (Additional file 1: Figure S2). The data represented as mean ± SEM. (**b**) IFN-γ and (**c**) TNF-α levels in cell culture supernatants of PBMCs and MYJ1633 were quantitatively measured using sandwich ELISA system. Significant differences between PBMC and MYJ1633 from 6 individuals were determined by Student’s t test. The mean value of each group is indicated with red bars

The expression of activating, natural cytotoxicity, and inhibiting receptors on CD16^+^CD56^+^ cells in MYJ1633 from 6 healthy donors was examined using flow cytometry. As shown in Fig. 3, the expression of activating receptors, NKG2D and DNAM-1, in the CD16^+^CD56^+^ MYJ1633 were 67.3 ± 8.4% and 67.3 ± 8.6%, respectively. The expression of natural cytotoxicity receptors, NKp44 and NKp46, were 32.9 ± 10.1% and 40.1 ± 8.4%, respectively. Finally, the expression of inhibiting receptor NKG2A in MYJ1633 was 46.6 ± 4.5% (Fig. 3a). Analyzed data using flow cytometry in CD16^+^CD56^+^ MYJ1633 are shown in (Additional file 1: Figure S2) and the expressions of activating and natural cytotoxicity receptors at 7 and 14 days after the initial culture are indicated in (Additional file 1: Figure S3).

### Studying cytokine release of MYJ1633 following ex vivo expansion

NK cells can take roles in immunoregulation by secretion of cytokines. Hence, we analyzed the release of IFN-γ and TNF-α, that are known to mediate the cytotoxic function of NK cells, before and after ex vivo expansion of MYJ1633. According to the data, the concentrations of IFN-γ and TNF-α in PBMC cultured media were 1.2 ± 0.02 ng/ml and 3.8 ± 0.1 ng/ml, respectively. However, after the ex vivo expansion of PBMC, the concentrations of IFN-γ and TNF-α increased to 529.1 ± 23.3 ng/ml and 520.4 ± 98.7 ng/ml, respectively (Fig. 3b and c).

### Investigating MYJ1633-mediated killing following ex vivo expansion of liver cancer cell lines

**Fig. 4:**
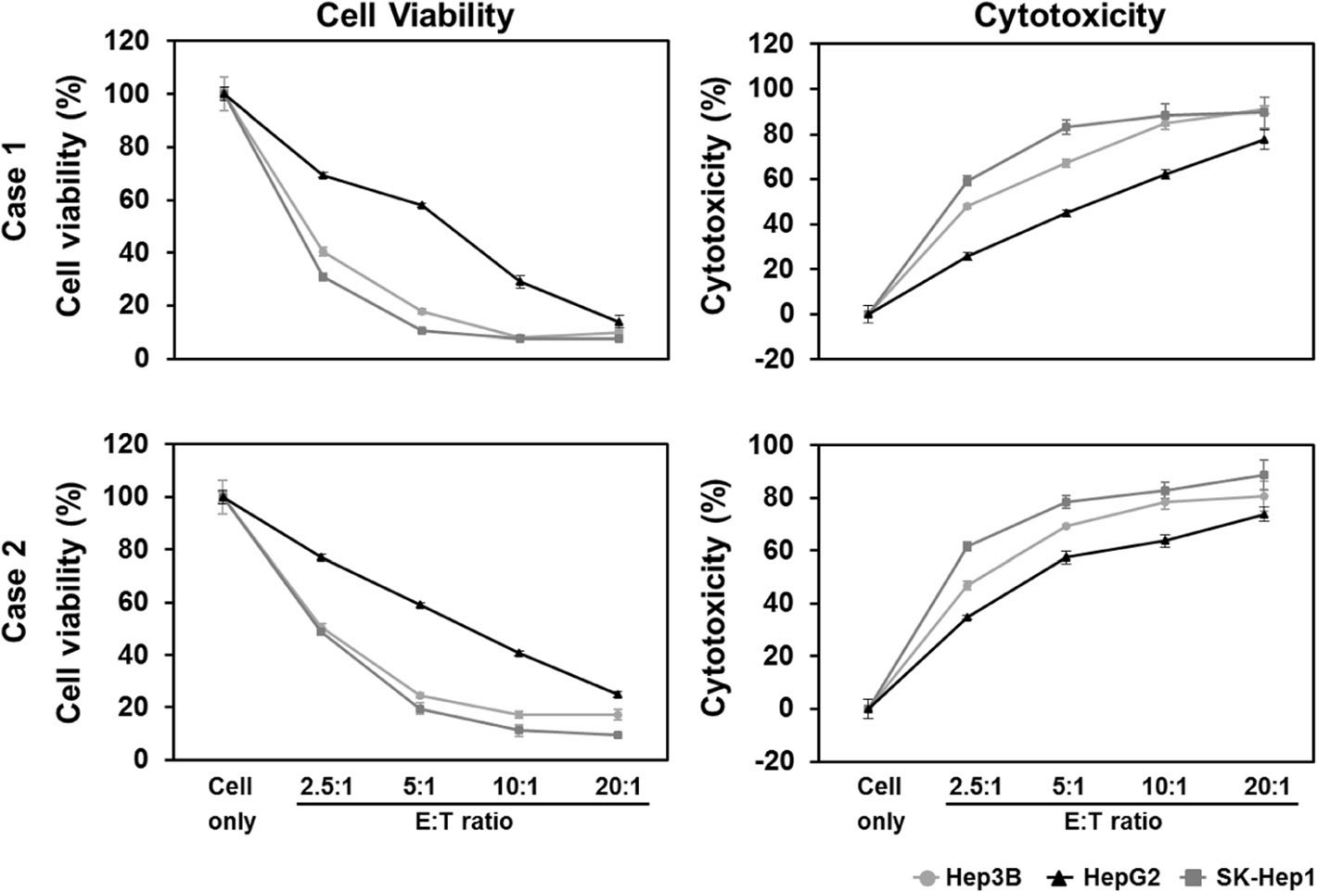
Cytotoxic potential of MYJ1633 against liver cancer cell lines. MYJ1633 was cultured with 3 different liver cancer cells for 24 h and the cell viability of cancer cells was measured using Ez-Cytox. The cytotoxicity of MYJ1633 against cancer cells was measured by LDH Cytotoxicity Detection Kit. Experiments were performed in triplicates. The data represented as mean ± SEM. E: effector (MYJ1633); T: target (cancer cells). Significant differences between control cancer cells and MYJ1633 co-cultured cancer cells were determined by Student’s t test and *p* values were below 0.0001 at all E:T ratio

The effect of MYJ1633 on the cell viability and the cytotoxicity of 3 different liver cancer cells (Hep3B, HepG2 and SK-Hep1 cells) were determined by WST-based cell viability assay and LDH-based cytotoxicity assay, respectively. As the E:T ratio increased the cell viability of all 3 liver cancer cells significantly decreased, while cytotoxicity on the cancer cells significantly increased. Although all 3 liver cancer cells were susceptible to the MYJ1633-induced cytotoxicity, SK-Hep1 was most sensitive to the MYJ1633, followed by Hep3B and HepG2 (Fig. 4).

### Investigating MYJ1633-mediated killing following ex vivo expansion of liver cancer animal models

**Fig. 5:**
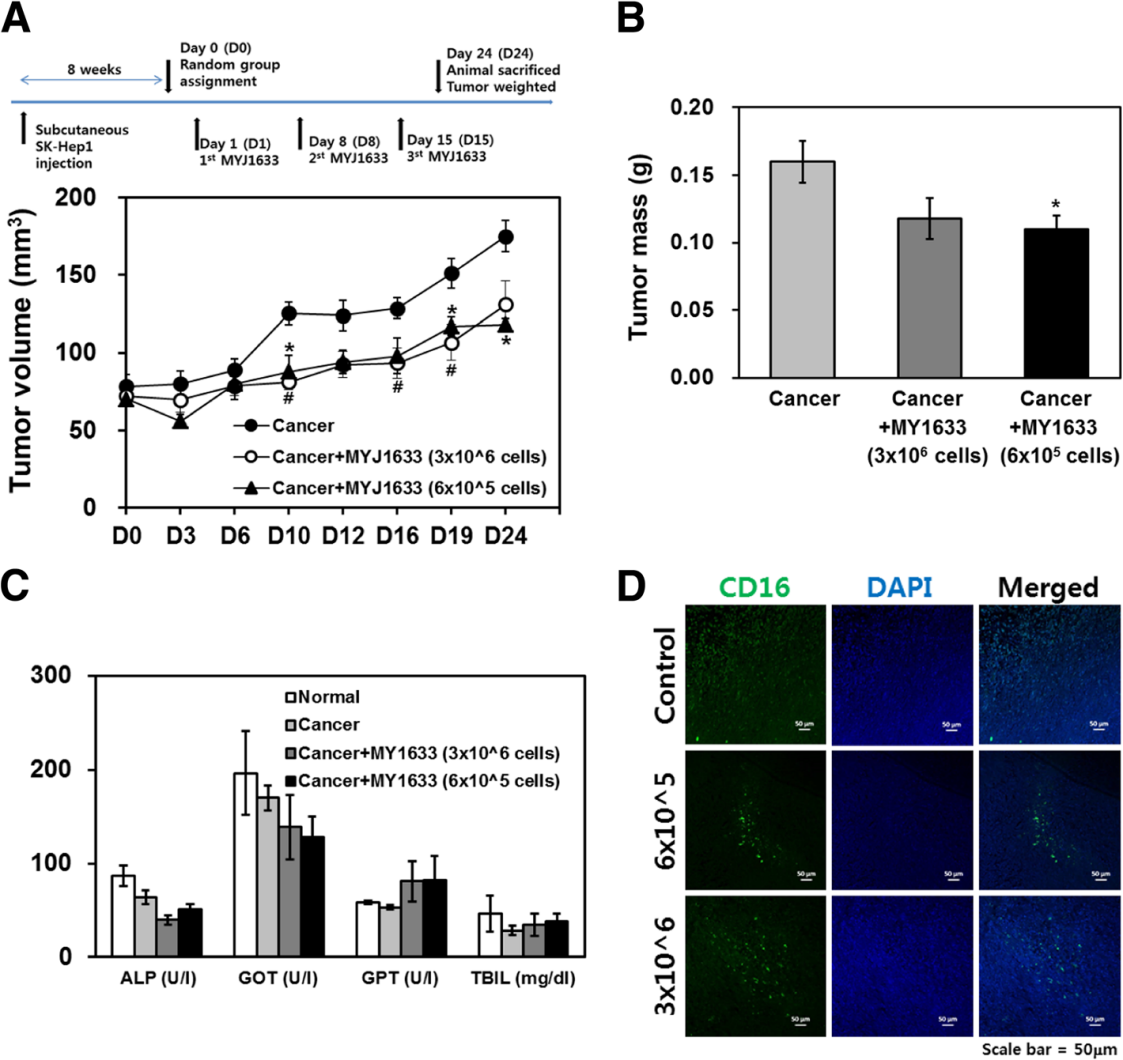
In vivo anti-tumor effect of MYJ1633 in a xenograft liver cancer animal model (**a**) The xenograft model was established using SK-hep1. Following random group assignment, the animals received 2 different concentrations of MYJ1633 (every 7 days, a total of 3 times) via tail vein infusion. The volume of tumor (width x width x length / 2) was monitored every 3–4 days for 24 days (*n* = 5 for each group). The data represented as mean ± SEM. *p < 0.05 and **p < 0.01. **b** Twenty four day after the group assignment, tumor mass was excised and weighted (n = 5 for each group). The data represented as mean ± SEM. *p < 0.05. **c** Blood chemistry analyzed at the end of the animal study (n = 5 for each group). ALP: alkaline phosphate, GOT: glutamate oxalacetate transaminase, GPT: glutamate pyruvate transaminase and TBIL: total bilirubin. Unit for each parameter is indicated in parenthesis. The data represented as mean ± SEM. **d** To identify human NK cells in tumor mass, immunohistochemial staining using human specific CD16 antibodies was conducted. CD16 positive cells were visualized using FITC-conjugated secondary antibodies and the nuclei were stained with DAPI

Two months after the subcutaneous injection of SK-Hep1, the volume of tumor reached approximately 80 mm^3^ in sufficient number of animals for random group. The volume of tumor in the MYJ1633 treated groups (81.3 ± 12.1 mm^3^ for 3 × 10^6^ of MYJ1633 group and 88.0 ± 25.2 mm^3^ for 6 × 10^5^ of MYJ1633 group) started to significantly differ from that of cancer group (125.3 ± 17.8 mm^3^) from day 10, and remained to be significantly smaller compared to that of cancer group to the end of the study (Fig. 5a). However, there was no significant difference between the group treated with 3 × 10^6^ of MYJ1633 and the group treated with 6 × 10^5^ of MYJ1633. Although there was no significant difference between the MYJ1633-treated groups, the tumor mass was significantly smaller in the MYJ1633-treated groups (0.11 ± 0.03 g and 0.12 ± 0.04 g for 3 × 10^6^ of MYJ1633 group and 6 × 10^5^ of MYJ1633 group, respectively) compared to that of cancer group (0.16 ± 0.04 g) (Fig. 5b). Upon gross examination of major organs (heart, liver and kidney) at the end of the study, no significant tissue damage was observed. Furthermore, chemical analysis of blood serum for ALP, GOT, GPT and TBIL indicated that there were no significant differences in those parameters detected among different groups (Fig. 5c). In inmmunohistochemical staining of the tumor mass using human CD16 specific antibodies, the number of human CD16 stained cells prominently increased in both MYJ1633 treated groups compared to the untreated control tumor group indicating the migration of MYJ1633 into the tumor mass (Fig. 5d).

## Discussion

It is important to secure effector cells with high cytotoxicity in large numbers for a successful adoptive cancer immunotherapy. One of our team’s long-term goals is to develop an effective autologous NK cell-based cell therapeutics which does not necessitate the use of NK cell purification. Furthermore, in our early trials, the expansion and activation of the purified NK cells were not as good as those of MYJ1633 in our experimental setting suggesting that certain factors derived from other type of cells such as T cells (eg. cytokines, growth factors, microRNAs, and/or vesicles containing them) could have played a beneficial role during the expansion and activation of MYJ1633. Although we are still trying to establish an effective expansion and activation protocol for purified NK cells as well, the major aim of the present study was to develop a new and simple ex vivo cell culture strategy to prepare large number of NK cells with elevated cytotoxicity from human PBMCs without an NK cell-sorting step.

To this end, PBMCs were isolated from the blood of healthy donors and cultured with selected cytokines (IL-2, IL-12, IL-18) for 2 weeks, because they may regulate the activation, proliferation and differentiation of leukocytes, including effector cells. (Fig. 1a). With the house-developed culture protocol, the total cell number increased 140-fold compared to the initially isolated PBMCs within 2 weeks (Fig. 1b). Flow cytometry using NK cell specific antibodies and T cell specific antibodies indicated that approximately 78% of the ex vivo-expanded and activated cells (MYJ1633) were CD16^+^/CD56^+^ cells (NK plus NKT cells) (Fig. 2a), and the majority of the T cells were CD3^+^CD8^+^ Tc cells (Fig. 2b). Further flow cytometric analysis indicated that ex vivo expansion of PBMCs in the presence of cytokines (IL-2, IL-12 and IL-18) and agonistic antibodies (CD16, CD56 and NKp46) increased the expression of activating receptors (NKG2D and DNAM-1) and cytotoxicity receptor (NKp44) (Additional file 1: Figure S3).

Regarding the cytokines use in this study, it has been reported that IL-2 regulates the activities of leukocytes and plays role in tolerance and immunity through via its direct effects on T cells [29]. It can also promote cell cycles and production of IFN-γ for proliferation and activation of NK cells [30, 31]. IL-12 plays an important role in the activities of T cells and NK cells by inducing production of IFN-γ and TNF-α [32]. In addition, it also mediates high cytotoxicity of NK cells and Tc cells [32]. Meanwhile, IL-2 induces the expression of two IL-12 receptors, IL-12R-β1 and IL-12R-β2, maintaining the expression of IL-12 signaling-related proteins in NK cells [33]. There is a report that activation of NK cells with IL-2 and IL-12 increased perforin binding and subsequent lysis of tumor cells [34]. In combination with IL-12, IL-18 induces cell-mediated immunity following infection, and NK cells and certain T cells secret IFN-γ or type II IFN after stimulation with IL-18. IL-12 and IL-18 exert striking synergistic activities for NK cell proliferation [35]. These cytokines are necessary but not sufficient for optimal proliferation of NK cells and Tc cells [36].

The cytolytic function of NK is controlled by a various activation and inhibitory receptors, and activating receptor (i.e., NKG2D) mediated target cell recognition induces the production of IFN- γ [37]. IFN-γ and TNF-α have been shown to be central in viral and tumor clearance [38–40]. NK cells express various activating and inhibitory receptors including NKG2D, DNAM-1 or killer immunoglobulin receptor (KIR), CD94 and natural cytotoxicity receptors (NKp30, NKp44 and NKp46) [41]. These receptors recognize major histocompatibility complex (MHC) class I and related molecules and cellular ligands, which can induce NK cell responses. Some of these receptors can also prevent activation of NK cells [41]. The cytotoxic activity of NK cells is regulated by the balance between activating and inhibitory signals derived from receptors expressed on the cell surface [42].

Our data demonstrated that MYJ1633 have anti-cancer potential against liver cancer cells both in vitro and in vivo (Figs. 4 and 5). In vitro study, MYJ1633-mediated cytotoxicity was elevated in all three liver cancer cell lines, namely Hep3B (hepatocellular carcinoma), HepG2 (hepatoblastoma) and SK-Hep1 (hepatocellular adenocarcinoma) cells with escalating E:T ratios. In in vivo study using SK-Hep1 to further validate the effectiveness of MYJ1633 against liver cancer, MYJ1633 treatment with 7 day-interval significantly suppressed tumor growth both in volumes and mass (Figs. 5a and b) without apparently significant change of blood chemistry (Fig. 5c). However, there was no significant difference between the two different concentrations of MYJ1633 in terms of suppressing tumor growth. At given experimental data given, it can be speculated that the number of MYJ1633, even the lower concentration, was sufficient to suppress tumor growth. Another possibility is that the duration of study might have not been long enough to see the different between groups. Our unpublished data using the same experimental design with longer study time (5 weeks) indicated that the difference between the groups tends to get larger as time increases. However, the study time could not be indefinitely elongated, since the overgrown cancer frequently causes the death of animal. Therefore, the dosage of NK cells may need to be optimized for each and every cancer model to clearly show the inter-group differences for future studies.

Although we did not conduct a systemic analysis for a time-dependent bio-distribution of NK cells for the present study, immunohistochemical analysis using human specific CD16 antibodies indicated that the injected MYJ1633 were migrated into the tumor mass (Fig. 5d). Considering the last MYJ1633 injection was 9 days prior to the sacrifice of animals, it can be assumed that the injected MYJ1633 survived and migrated into the tumor mass at the least for up to 9 days. Recently, similar results have been reported by other groups [43]. According to their study, intravenously injected NK cells were initially concentrated in the lung and kidney, but rapidly disappeared at 4 h after the injection in non-tumor-bearing animals. On the other hand, in tumor (MDA-MB-231, triple negative breast cancer) bearing-animals, NK cells migrated to the tumor mass and persisted for up to 7 days.

One of the limitations of the present study is that, by using athymic animals, our study was unable to take account of the effect of T cells on the bio-activity of MYJ1633 in vivo. For example, using a syngeneic tumor mouse model would have been definitely ideal for validating the effect of MYJ1633 in a more clinical-relevant setting. Since our team is focused on autologous NK cell-based therapeutics, we also envision an animal model system where collecting and expanding NK cells from the PBMCs of syngeneic mice. Taken together, our ex vivo-expansion protocol is very effective for potentiating the cytotoxicity of NK cells and Tc cells (MYJ1633) and these results suggest possibility of clinical application of MYJ1633 for liver cancer immunotherapy.

## Conclusions

In conclusion, we developed and empirically verified a new and simple ex vivo-expansion protocol using IL-2, IL-12, IL-18, CD16, CD56 and NKp46 for preparing high ratio of NK cells in effector cells (MYJ1633) and demonstrated their cytotoxicity against liver cancer in vitro and in vivo. These results provide a meaningful experimental and theoretical base for future progression of NK cell-mediated anti-tumor immunotherapy.

## Additional file

Additional file 1: **Figure S1.** NK, NKT, and T cell composition of MYJ1633. (A) Composition of NK cells (CD3^−^CD16^+^CD56^+^), NKT cells (CD3^+^CD16^+^CD56^+^), and T cells (CD3^+^CD16^−^CD56^−^) in freshly isolated PBMCs and MYJ1633 (B) Proportion of helper T cells (Th cells; CD4^+^) and cytotoxic T cells (Tc cells; CD8^+^) among CD3^+^ cells of MYJ1633. **Figure S2.** Expression of activating, natural cytotoxicity and inhibiting receptors on CD16^+^CD56^+^ cells of MYJ1633**.** Using 14 day cultured MYJ1633 from 6 individuals, the expression of activating receptors (NKG2D and DNAM-1), natural cytotoxicity receptors (NKp44 and NKp46), and inhibiting receptor (NKG2A) was determined by flow cytometry. **Figure S3.** Time-dependent expression change of activating and natural cytotoxicity receptors on CD16^+^CD56^+^ cells of MYJ1633. The expression of activating receptors and natural cytotoxicity receptors of 7 day cultured and 14 day cultured MYJ1633 from 6 individuals was examined by flow cytometry. The data represented as mean ± SEM. (PDF 839 kb)

## Data Availability

The datasets used and/or analyzed during the current study available from the corresponding author on reasonable request.

## Abbreviations

IFN: Interferon
NKL: Natural killer cell-enriched lymphocytes
PBMC: Peripheral blood mononuclear cells
TNF: Tumor necrosis factor
