# Supplementary material for: Cytotoxic effects of ex vivo-expanded natural killer cell-enriched lymphocytes (MYJ1633) against liver cancer
Source: BMC Cancer. 2019 Aug 19;19:817. doi: 10.1186/s12885-019-6034-1 (PMC6700835; doi:10.1186/s12885-019-6034-1)
Supplement: Supplementary file 1 — Figure S1. NK, NKT, and T cell composition of MYJ1633. (A) Composition of NK cells (CD3−CD16+CD56+), NKT cells (CD3+CD16+CD56+), and T cells (CD3+CD16−CD56−) in freshly isolated PBMCs and MYJ1633 (B) Proportion of helper T cells (Th cells; CD4+) and cytotoxic T cells (Tc cells; CD8+) among CD3+ cells of MYJ1633. Figure S2. Expression of activating, natural cytotoxicity and inhibiting receptors on CD16+CD56+ cells of MYJ1633. Using 14 day cultured MYJ1633 from 6 individuals, the expression of activating receptors (NKG2D and DNAM-1), natural cytotoxicity receptors (NKp44 and NKp46), and inhibiting receptor (NKG2A) was determined by flow cytometry. Figure S3. Time-dependent expression change of activating and natural cytotoxicity receptors on CD16+CD56+ cells of MYJ1633. The expression of activating receptors and natural cytotoxicity receptors of 7 day cultured and 14 day cultured MYJ1633 from 6 individuals was examined by flow cytometry. The data represented as mean ± SEM. (PDF 839 kb) [file 12885_2019_6034_MOESM1_ESM.pdf]

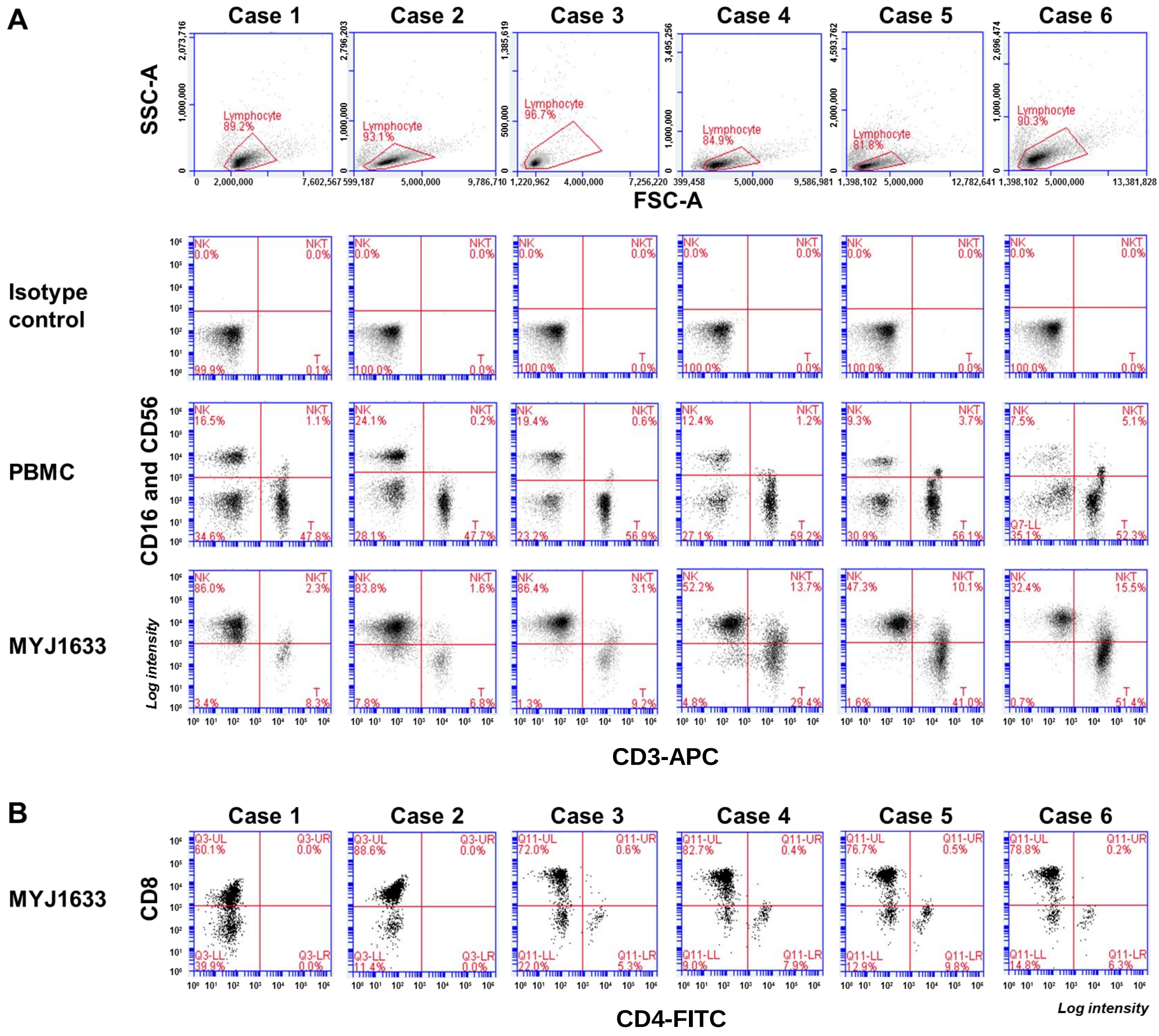

Figure S1. NK, NKT, and T cell composition of MYJ1633. (A) Composition of NK cells ($CD3^-CD16^+CD56^+$), NKT cells ($CD3^+CD16^+CD56^+$), and T cells ($CD3^+CD16^-CD56^-$) in freshly isolated PBMCs and MYJ1633 (B) Proportion of helper T cells (Th cells; $CD4^+$) and cytotoxic T cells (Tc cells; $CD8^+$) among $CD3^+$ cells of MYJ1633.

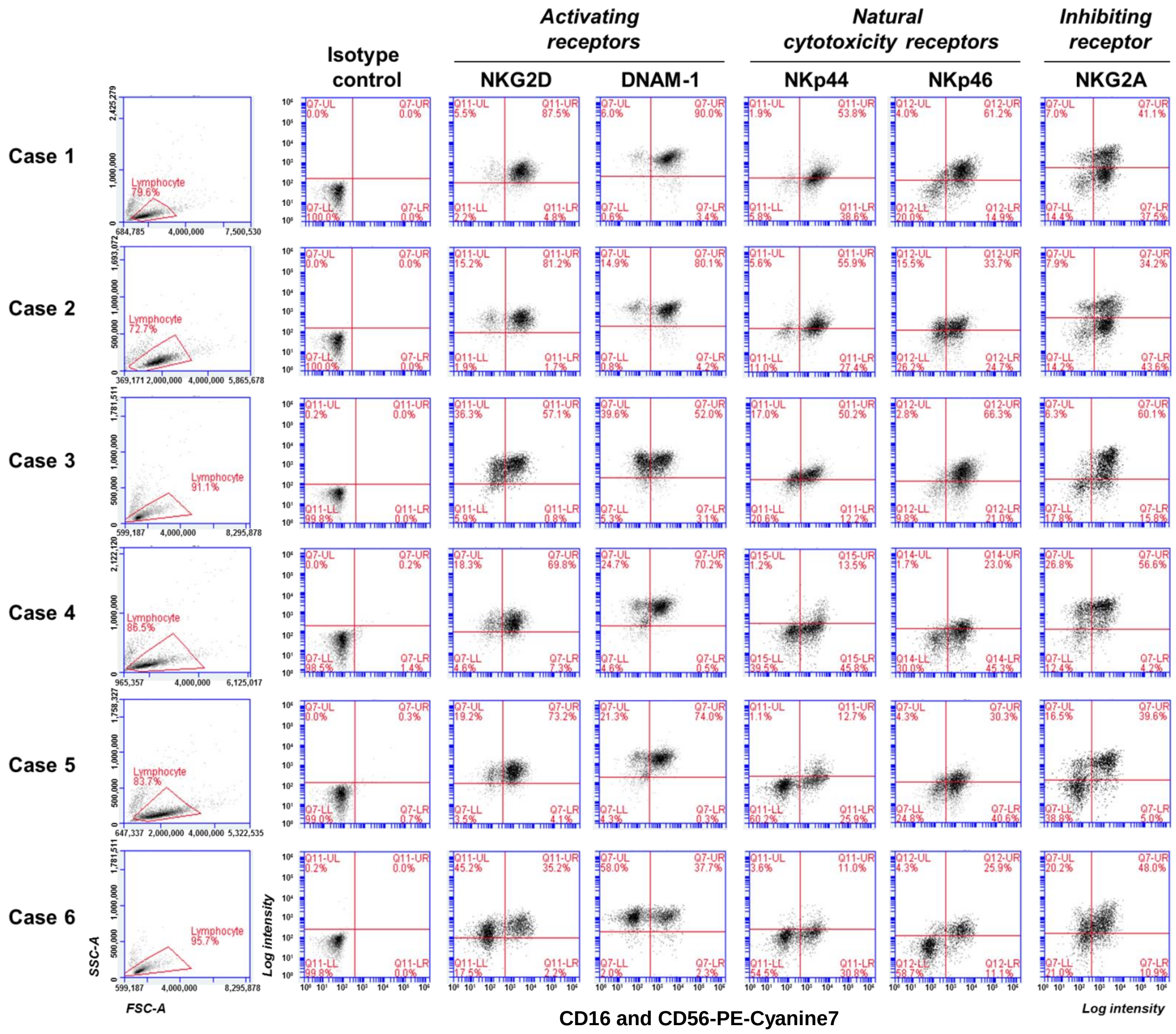

Figure S2. Expression of activating, natural cytotoxicity and inhibiting receptors on CD16⁺CD56⁺ cells of MYJ1633. Using 14 day cultured MYJ1633 from 6 individuals, the expression of activating receptors (NKG2D and DNAM-1), natural cytotoxicity receptors (NKp44 and NKp46), and inhibiting receptor (NKG2A) was determined by flow cytometry.

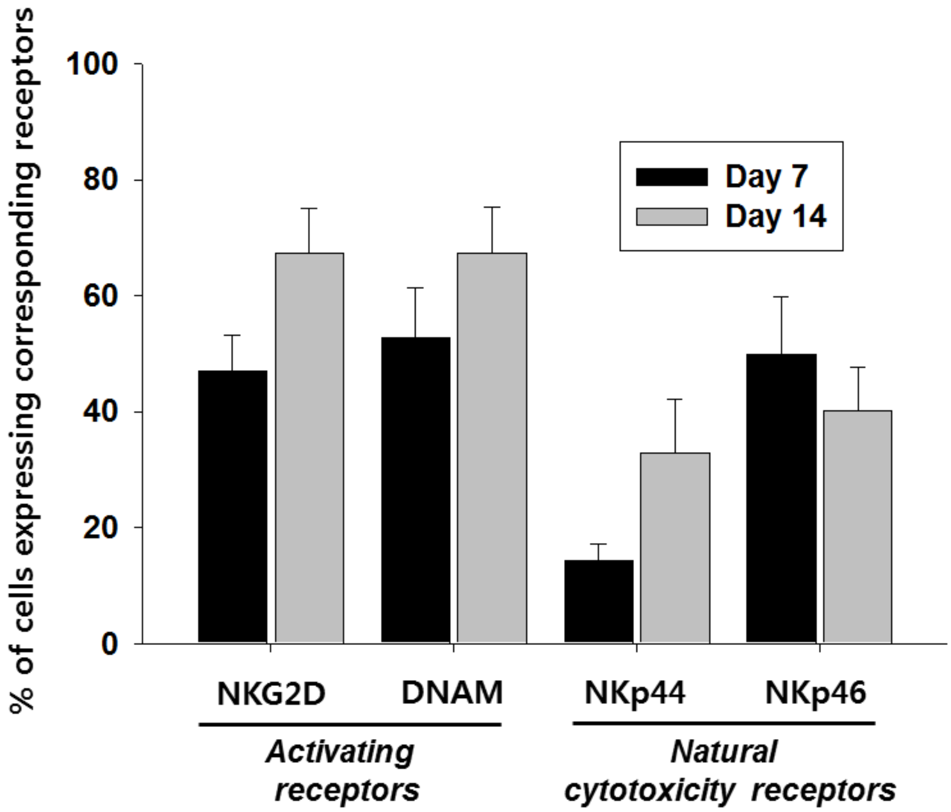

Figure S3. Time-dependent expression change of activating and natural cytotoxicity receptors on CD16⁺CD56+ cells of MYJ1633. The expression of activating receptors and natural cytotoxicity receptors of 7 day cultured and 14 day cultured MYJ1633 from 6 individuals was examined by flow cytometry. The data represented as mean ± SEM.
